# Clinical, Laboratory, and Molecular Epidemiology of an Outbreak of Aseptic Meningitis Due to a Triple-Recombinant Echovirus in Ashburton, New Zealand

**DOI:** 10.3390/v14040658

**Published:** 2022-03-22

**Authors:** Meik Dilcher, Julia C. Howard, Simon C. Dalton, Trevor Anderson, Richard T. Clinghan, Anja M. Werno

**Affiliations:** 1 Microbiology Department, Virology/Serology Section, Canterbury Health Laboratories, Christchurch 8011, New Zealand; julia.howard2@cdhb.health.nz (J.C.H.); trevor.anderson@cdhb.health.nz (T.A.); anja.werno@cdhb.health.nz (A.M.W.); 2 Department of Infectious Diseases, Christchurch Hospital, Christchurch 8011, New Zealand; simon.dalton@cdhb.health.nz; 3 General Medicine Department, Ashburton Hospital, Ashburton 7700, New Zealand; rclinghan@outlook.com

**Keywords:** enterovirus, meningitis, molecular epidemiology, recombination

## Abstract

Here, we describe a small enterovirus outbreak including nine cases of aseptic meningitis in a New Zealand hospital in 2017. Most patients had a lymphocytic predominance in the CSF, their length of stay was short, and there were no paediatric cases or ICU admissions. VP1 genotyping revealed that the outbreak was caused by an echovirus E30 strain closely related to strains reported from the US, UK, Brazil, and Denmark. They all form a separate cluster within lineage “h”, which leads to the proposal of establishing a new lineage tentatively named “j” for this group of echovirus E30 strains. However, whole genome sequencing and reference mapping to echovirus E30 sequences showed very poor mapping of reads to the 3′ half of the genome. Further bioinformatic analysis indicated that the causative agent of this outbreak might be a mosaic triple-recombinant enterovirus composed of echovirus E6, echovirus E11, and echovirus E30 genome segments.

## 1. Introduction

Enteroviruses are viral pathogens causing a wide spectrum of diseases in humans ranging from mild common cold-like symptoms, hand foot and mouth diseases, acute haemorrhagic conjunctivitis, myocarditis, acute flaccid paralysis (including poliomyelitis), to fatal aseptic meningitis or encephalitis [1]. National enterovirus surveillance data provided by the ESR in Wellington since 1995 show alternating annual patterns of enterovirus activity, with years with very low activity followed by years with high activity frequently causing outbreaks (https://surv.esr.cri.nz/virology/virology_annual_report.php, accessed on 12 March 2022). Main drivers of enterovirus outbreaks in New Zealand were Coxsackie virus A24 (1997), Echovirus E33 (2000), Echoviruses E13 and E30 (2001/2002), Enterovirus type 68 (2010), and Coxsackie virus A6 (2013). In 2016 and 2017, the most predominant enterovirus serotypes were Coxsackie virus A6, Echovirus E18, and Echovirus E30 (22 laboratory-confirmed cases).

We became aware of an unusual clustering of enteroviral meningitis cases caused by Echovirus E30 in Ashburton, New Zealand over several months in 2017. Ashburton is a small district with a population of about 32,400 people. Echovirus E30 is a member of the enterovirus genus and belongs to human enterovirus species B (HEV-B). It has been associated with large outbreaks of aseptic meningitis in adults and children in the community [2] as well as in families [3] and sports teams [4]. It was decided to undertake genotyping and further genomic characterization to determine how closely related the cases were and to establish phylogenetic relationships to other global Echovirus E30 strains.

## 2. Materials and Methods

Samples: Cerebrospinal fluid (CSF) samples from patients with aseptic meningitis were collected in Ashburton Hospital between August and November 2017.

RNA extraction: Nucleic acids were extracted with the standard EasyMag (bioMérieux, Marcy-l’Étoile, France) generic 2.0 extraction protocol (400 µL sample input, 50 µL elution volume).

Real-time PCR: Real-time RT-PCR molecular diagnostic testing of the 9 CSF samples was completed with an in-house meningitis/encephalitis PCR panel targeting herpes simplex virus 1/2 plus varicella zoster virus in pool 1, enterovirus, and parechovirus in pool 2 [5] and *Neisseria meningitidis* in pool 3.

Genotyping: VP1 gene genotyping was performed using a 2-step nested RT (reverse transcriptase)-PCR method described by Nix et al. [6], with modification of the procedure using random hexamer primers instead of degenerated primers in the RT reaction step. During the nested PCR, a 384 bp partial fragment of the enterovirus VP1 capsid gene is amplified that correlates with the serotype determined by antigenic methods. The synthesis of cDNA (step-1) was carried out in a 10 µL reaction volume containing 5 µL or RNA, 0.8 µL dNTPs (10 mM each), 2.0 µL 5× AMV Buffer (Roche, Basel, Switzerland), 0.2 µL random hexamers (50 µM), 0.5 µL RNase out (Invitrogen, Waltham, MA, USA), 0.25 µL AMV RT enzyme (Roche, Basel, Switzerland), and 1.25 µL water. The reaction was incubated for 20 min at 42 °C and the RT enzyme was inactivated by incubation for 5 min at 95 °C. Round 1 PCR (step-2) was carried out in a 50 µL reaction volume containing 10 µL cDNA, 5 µL 10× Roche FastStart Taq Buffer (with MgCl_2_), 0.8 µL dNTPs (10 mM each), 1 µL 224 primer (50 µM), 1 µL 222 primer (50 µM), 0.5 µL FastStart Taq polymerase (Roche, Basel, Switzerland), and 31.7 µL water. The reaction was incubated for 10 min at 95 °C to activate the Taq polymerase. This was followed by 40 cycles of 95 °C for 30 s, 42 °C for 30 s, and 60 °C for 45 s. The programme finished with a final elongation at 72 °C for 5 min. Round 2 PCR was carried out in a 25 µL reaction volume containing 1 µL round 1 PCR product, 2.5 µL 10× Roche FastStart Taq Buffer (with MgCl_2_), 0.4 µL dNTPs (10 mM each), 0.4 µL AN89 primer (50 µM), 0.4 µL AN88 primer (50 µM), 0.25 µL FastStart Taq polymerase (Roche, Basel, Switzerland), and 20.05 µL water. The reaction was incubated for 10 min at 95 °C to activate the Taq polymerase. This was followed by 40 cycles of 95 °C for 30 s, 60 °C for 20 s, and 72 °C for 20 s. A final elongation at 72 °C was performed for 5 min. Round 2 PCR amplicons (384 bp region of the VP1 gene) were visualised by agarose gel electrophoresis. Sanger sequencing was performed using a BigDye Terminator v3.1 ready reaction cycle sequencing kit on an ABI Prism 3130xl automated sequencer (Applied Biosystems/Thermo Fisher Scientific, Waltham, MA, USA) with primers AN232 and AN233 in separate reactions.

Unbiased whole genome sequencing: We were able to isolate sample 17WQ2027G collected on the 20 August 2017 on MRC-5 cells. Host cells were lysed by freeze–thawing, and host nucleic acids were depleted using benzonase (Sigma-Aldrich/Merck, St. Louis, MO, USA) treatment for 2 h at 37 °C. The benzonase was inactivated by the addition of 0.5 M EDTA (final conc. 6.25 mM), and viral nucleic acids were extracted using EasyMag (bio>Mérieux, Marcy-l’Étoile, France) generic 2.0 extraction protocol and quantified via a Qubit RNA HS Assay kit (Thermo Fisher Scientific, Waltham, MA, USA). First-strand cDNA synthesis was carried out for 1 h at 60 °C with Transcriptor Reverse Transcriptase (Roche, Basel, Switzerland) using random hexamer primers. The NEBNext Ultra II Non-Directional RNA Second Strand Synthesis Module (NEB, Ipswich, MA, USA) was used for second-strand cDNA synthesis at 16 °C for 1 h. Double-stranded cDNA was purified using AmpureXP beads (Beckman Coulter, Brea, CA, USA) and quantified using Qubit High Sensitivity DNA assay (Thermo Fisher Scientific, Waltham, MA, USA). Barcoded NGS libraries were generated following the Nextera DNA Flex protocol (Illumina, San Diego, CA, USA). The sequencing was performed on MiSeq using Reagent Kit v2 (300 cycles) micro flow cell.

Bioinformatic analysis: Maximum-likelihood phylogenetic analysis of the partial VP1 genotyping sequences and whole genome sequences was carried out using MEGA v5.1 after ClustalW alignment, which was inferred by bootstrapping for 500 replicates. De novo genome assembly was performed with SPAdes v3.11.0 [7] and reference mapping was performed with CLC Genomics Workbench v20.0.2 (Qiagen, Hilden, Germany). Approximately-maximum-likelihood phylogenetic analysis of the whole genome enterovirus sequences was completed using FastTree v2.1.10 [8] after multiple sequence alignment based on fast Fourier transform with MAFFT v7.310 [9]. The whole genome phylogenetic tree was visualised with FigTree v1.4.4. A Simplot BootScan v. 3.5.1 [10] analysis was applied to a ClustalW alignment of the de novo assembled enterovirus genome MW586892 (strain NZ/Ashburton/17WQ2027G/2017-08-20) with echovirus E6 (isolate E6/P735/2013/China, accession number KP289439), echovirus E11 (strain 520k-YN-CHN-2010, accession number KP294524) and echovirus E30 (strain 14-397 collected 2013, accession number KY888274) sequences. BootScan analysis was performed using the Kimura (2-parameter) distance model with 500 bootstrap replicates based on a neighbour-joining tree.

## 3. Results

### 3.1. Clinical Findings

Nine patients were hospitalised over a 13-week period spanning August to November in 2017. The age range was 16 to 55 years (55.6% male) and included a husband and wife. The median length of hospital stay was one day, and none required ICU admission. Presenting symptoms were headache (100%), photophobia (88.9%), fever (88.9%), neck stiffness (66.7%), and nausea and/or vomiting (77.8%). Out of all the patients, 7/9 had a CT head, of which all were normal bar one, which showed mild mucosal changes of the sinuses.

### 3.2. Laboratory Findings

**Figure 1:**
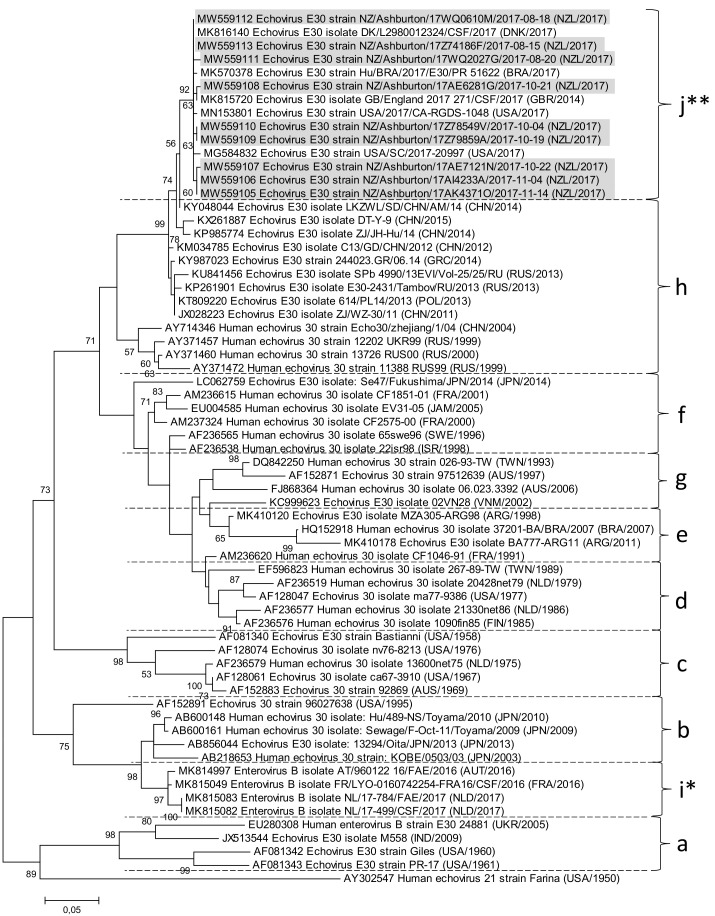
Maximum-likelihood phylogenetic tree (MEGA) after ClustalW alignment of partial VP1 sequences (331 nt), inferred by bootstrapping for 500 replicates. Genbank accession numbers for Ashburton Echovirus E30 samples highlighted in grey: MW559105, MW559106, MW559107, MW559108, MW559109, MW559110, MW559111, MW559112, and MW559113. Lineages are identified based on the publication by Miyoshi et al. 2019 [11]. *: tentative “lineage i” as suggested by Gámbaro et al. 2021 [12]. **: tentative “lineage j” as suggested in this publication. Echovirus E30 prototype strain Bastianni (AF081340) is included in “lineage c”. Human echovirus 21 strain Farina (AY302547) was used as an outgroup to root the tree.

All samples had raised white cell count (WCC) in the cerebrospinal fluid (CSF) (mean = 290, range 83–905), 8/9 had a lymphocytic predominance, CSF protein was 0.32–1.24 g/L, and CSF glucose was 3.1–3.9 mmol/L. Peripheral WBC was 4.4–13.0. The CRP range was 4–63 mg/L (for details, see Supplementary Table S1). All CSFs showed no bacterial growth using standard bacteriological methods. Diagnosis of enteroviral meningitis of CSF samples was made using a meningitis/encephalitis PCR panel. All samples had medium to high Ct values for enterovirus (MW559105: Ct 33, MW559106: Ct 30, MW559107: Ct 36, MW559108: Ct 36, MW559109: Ct 34, MW559110: Ct 37, MW559111: Ct 35, MW559112: Ct 32, MW559113: Ct 35). In view of the temporal and spatial clustering of cases, genotyping was undertaken, which revealed 9/9 samples to have 98% to 99% sequence identity to VP1 sequences from echovirus E30 sequences after Blastn analysis. A ClustalW alignment and maximum-likelihood phylogenetic tree of the partial VP1 sequences (quality-trimmed 331 bp regions) revealed the closest relationship of the Ashburton samples (grey) to echovirus E30 samples from the USA, Brazil, and Denmark from 2017 as well as the UK and from 2014 (Figure 1).

**Figure 2:**
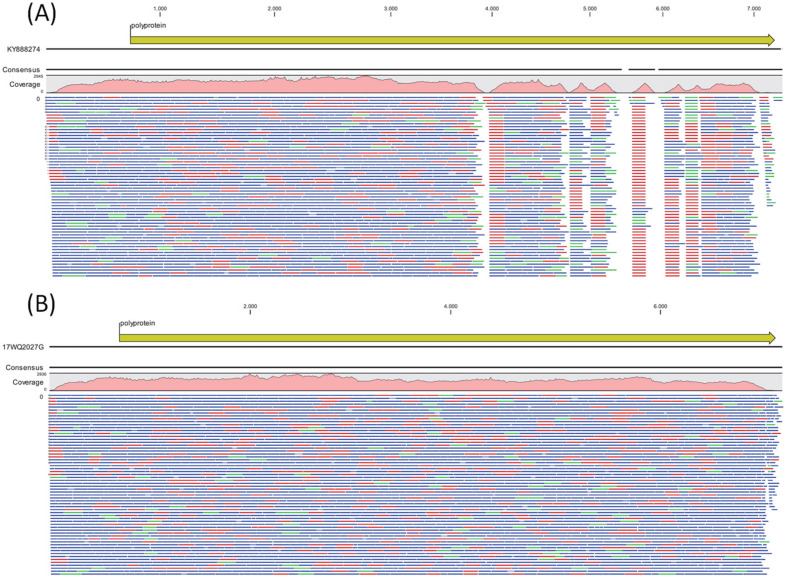
(**A**) Reference mapping of obtained NGS reads against Echovirus E30 strain 14–397, accession number KY888274 reveals good read coverage for the 5′ half of the genome but gaps in the 3′ half of the genome. (**B**) Mapping of reads against de novo assembled genome of MW586892 strain NZ/Ashburton/17WQ2027G/2017-08-20 showing good coverage of reads across the whole genome. Forward reads = green, reverse reads = red, paired reads = blue. Only a proportion of mapped reads are shown.

All show the closest clustering to samples from “lineage h” as determined by comparison with strains described in the publication by Miyoshi et al. 2020 [11]. However, they can be clearly discriminated from “lineage h” by forming a separate clade, which we suggest being tentatively named “lineage j”, since Gámbaro et al. 2021 [12] have already suggested to tentatively name a sub-cluster of lineage b “lineage i”. Within the 331 bp genotyping window, the nine Ashburton samples showed slight differences in six positions (with 98.79% similarity). This is probably attributable to different sampling times covering a period of 13 weeks. One sample (strain NZ/Ashburton/17WQ2027G/2017-08-20) could be isolated in MRC-5 cell culture and was subjected to host depletion via benzonase treatment, RNA extraction, first and second-strand cDNA synthesis with random hexamer primers and Illumina whole-genome sequencing. Almost the complete genome (7393nt/7414nt compared to E30 reference strain Bastianni KY888275) could be assembled with an average coverage of 1824x, containing the complete coding sequence for the polyprotein and parts of the 5′ and 3′ NCR’s (GenBank accession number MW586892). Blastn analysis of the whole genome revealed 89% sequence identity to other echovirus E30 genomes (e.g., strain 14–397, accession number KY888274) submitted until 2017, whereas Blastp analysis of the polyprotein sequence showed 98% identity to other echovirus E30 polyprotein sequences. Interestingly, reference mapping to other echovirus E30 genomes (e.g., strain 14–397 collected in Germany in 2013, accession number KY888274, Figure 2A) yielded good coverage for the 5′ half of the genome, but mapping gaps in the 3′ half of the genome, whereas mapping of reads against the de novo assembled genome of MW586892 strain NZ/Ashburton/17WQ2027G/2017-08-20 showed good coverage across the whole genome (Figure 2B).

**Figure 3:**
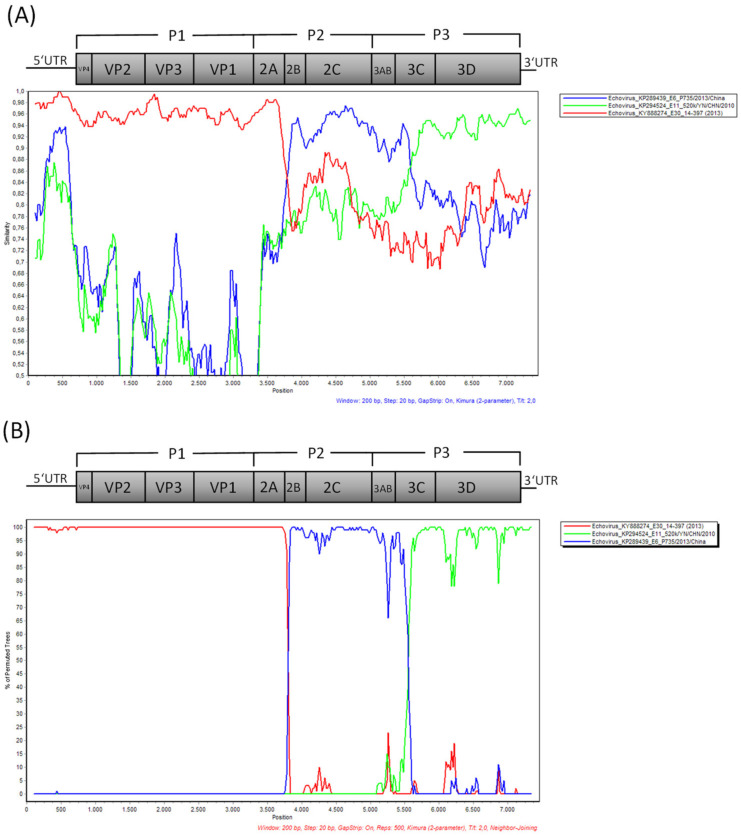
Whole genome Simplot BootScan analysis with the query sequence of strain NZ/Ashburton/17WQ2027G/2017-08-20 (accession number MW586892) compared to Echovirus E6 (accession number KP289439), E11 (accession number KP294524), and E30 (accession number KY888274) reference sequences. (**A**) Similarity plot analysis, (**B**) Bootscanning analysis on the putative recombinant strain and its parental sequences. The enterovirus genomic organization is shown in the top.

A Simplot BootScan analysis (Figure 3) indicated that the Ashburton echovirus genome could be a mosaic triple-recombinant of echovirus E30 (nucleotides approximately 0 to 3800 representing the P1 region encoding the capsid proteins as well as a short part of the P2 region encoding the 2A gene with overall identity of 96% to echovirus E30 KY88274), echovirus E6 (nucleotides approximately 3800 to 5600 representing the remainder of the P2 region and the first part of the P3 region encoding non-structural proteins finishing within the 3C gene, showing 94% identity to echovirus E6 KP289439), and echovirus E11 (nucleotides approximately 5600 to 7393, representing the remainder of the P3 region encoding non-structural proteins including the RNA-dependent RNA polymerase 3D, 94% identity with echovirus E11 KP294524).

**Figure 4:**
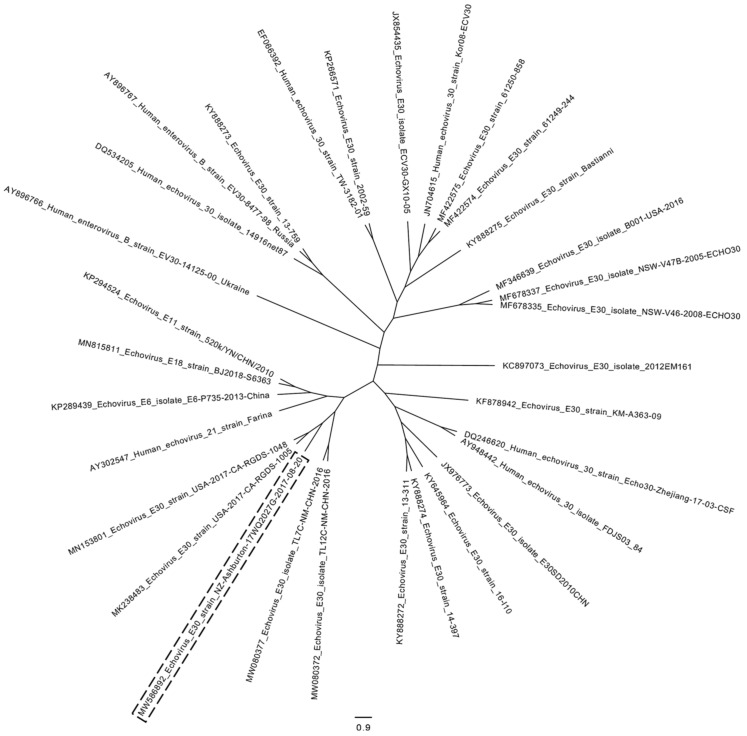
Maximum-likelihood phylogenetic tree (FastTree) of echovirus whole genome sequences after multiple sequence alignment based on fast Fourier transform (MAFFT) visualised with FigTree. Ashburton Echovirus E30 accession number MW586892 indicated with a dashed box in a cluster of other closely related E30 echoviruses from California and China. Echovirus E6, E11, E18, and echovirus 21 strain Farina are included as an outgroup.

This could explain the poor mapping result of the 3′ half of the genome to other echovirus E30 genomes available in GenBank in 2017. However, the E30, E6, and E11 echoviruses used for the Simplot BootScan recombination analysis are not prototype strains and therefore could be recombinants in the 3′ half of the genomes themselves. An additional Simplot BootScan analysis of an alignment of the NZ Echovirus E30 MW586892 with available whole genome sequences of the enterovirus prototype strains described in [12] is presented in Supplementary Figure S1. Here, the 5′ half of the genome showed the highest similarity to E30 prototype strain Bastianni, and a signal of recombination appeared between the P1 and P2 region, but none of the prototype strains showed a defined similarity over a longer sequence stretch in the 3′ half of the genome. Interestingly, in a more recent Blastn analysis, the 3′ half of the genome of MW586892 (nucleotides approximately 3800 to 7393) shows a nucleotide similarity of 95% to E18 echoviruses collected in China in 2018, e.g., MN815811. A Simplot BootScan analysis of the whole genome sequence of these E18 echoviruses aligned to E6 and E11 whole genome sequences yields a good match of the first part of the 3′ half to echovirus E6, with the second part of the 3′ end matching to echovirus E11 in a similar pattern to that seen in Figure 3B (data not shown). This indicates that the 3′ ends of these E18 echoviruses might also be derived from recombination events with E6 and E11 echoviruses or a recombination event with E30 echoviruses of lineage “j” such as the one described in this manuscript. To further prove the intertypic recombination in the 3′ half of the genome of the NZ Echovirus E30 MW586892, we generated additional phylogenetic trees based on the P1 (nt 726–3339) as well as P2 (nt 3340–5039) and P3 (nt 5040–7167) coding regions including all enterovirus prototype strains described in [12] (see Supplementary Figure S2). Here, as expected, the P1 capsid region showed close clustering of MW586892 with E30 prototype strain Bastianni (AF311938). The P2 non-capsid region clustered closer to E12 prototype strain Travis (X79047), whereas the P3 non-capsid region clustered closest to enterovirus B prototype strain EVB86 (AY843304). However, E30 prototype strain Bastianni (AF311938) is part of the same phylogentic clade, indicating some similarity to E30 in the P3 region. Interestingly, no clustering with E6 and E11 prototype strains could be observed for the P2 and P3 regions. In November 2018 and July 2019, two echovirus E30 genomes (accession numbers MK238483 and MN153801) from a large meningitis outbreak in California were submitted to GenBank that show 99% sequence identity to the Ashburton echovirus E30 (query coverage 96%). In addition, in October 2020, two echovirus whole genome sequences (MW080372 and MW080377) from an outbreak of aseptic meningitis in inner Mongolia were submitted to GenBank that show 96% sequence identity to the Ashburton echovirus E30 (query coverage 99%). All these epidemic echovirus strains from the US and China form a separate cluster with the Ashburton echovirus (labelled with a dashed box) in a maximum-likelihood phylogenetic tree in Figure 4, indicating the emergence of a new epidemic lineage.

## 4. Discussion

There is little current data about which enteroviruses are circulating in New Zealand. After a cluster of aseptic meningitis cases appeared in Ashburton, New Zealand, we decided to analyse the cases via VP1 genotyping. This revealed that the outbreak was caused by echovirus E30, with the closest clustering to strains from lineage h. However, since the reported echoviruses closely cluster with other echovirus E30 strains from 2014 and 2017 detected in the US, UK, Brazil, and Denmark, which form a separate distinguishable clade in the phylogenetic VP1 tree, we suggest grouping them into a newly established lineage tentatively named “j”. Echovirus E30 has been associated with outbreaks of aseptic meningitis globally and has the potential to cause very large outbreaks [13,14]. In keeping with previous reports of outbreaks, most of the patients had a lymphocytic predominance in the CSF, length of stay was short, and there were no ICU admissions. Many of the cases reported recent contact with unwell children, but surprisingly, there were no paediatric cases in this cluster.

Our whole genome data analysis indicates that this newly discovered echovirus might be a mosaic triple-recombinant between echovirus E30, echovirus E6, and echovirus E11, which were most probably derived via intertypic/heterotypic homologous recombination during co-infection. However, the E6 and E11 strains that show the highest homology in the P2 and P3 regions could themselves be recombinant viruses as they were probably only classified based on their VP1 sequence. A comparison with enterovirus reference sequences showed no matches to E6 and E11 protype strains in the 3′ half of the genome (see Supplementary Figures S1 and S2). Therefore, which parental enterovirus prototype strain might have contributed the P2 and P3 genomic regions remains elusive. In the recent literature, mainly double-recombinant enteroviruses are described, e.g., between echoviruses E30 and E6 [15] or echovirus E13 and Human Coxsackievirus B3 [12]. Epidemic echoviruses seem to appear and disappear in cycling patterns [11] with the emergence of new recombinant echovirus E30 strains every 3–5 years [16]. This might be a way to evade herd immunity in the population. Recombination of the modular genomes of enteroviruses is an evolutionary mechanism for rapidly acquiring regions of novel sequences and is implicated for example in the emergence of novel neuropathogenic variants of poliovirus [17] or the emergence of circulating vaccine-derived polioviruses composed of mosaic genomes of poliomyelitis vaccine virus and sequences of non-poliovirus enteroviruses [18]. However, the exact molecular recombination mechanism remains relatively poorly understood [17,18].

In addition, our results show that genotyping based on partial VP1 Sanger sequences might not be sufficient to characterise echovirus outbreaks, whereas whole genome sequence data provide much more detailed information, especially in regard to recombination events. Since the VP1 genotyping window is located in the 5′ half of the genome, it will always show the highest homology to a certain strain, such as in this case echovirus E30, even if the remainder of the genome has undergone recombination events with other echovirus strains or other enterovirus species. Interestingly, closely related sequences of this new triple-recombinant echovirus variant have also been detected in big aseptic meningitis outbreaks in California and Mongolia, implicating that this new variant has the potential to cause aseptic meningitis epidemics possibly due to new antigenic properties or altered replication or pathogenicity. Therefore, continuous molecular epidemiological surveillance via whole genome sequencing could provide a tool to better understand the true complexity and evolution of enterovirus genomes and their association with outbreaks and certain clinical manifestations.

## Data Availability

The data presented in this study are available within the article. Sequences have been submitted to GenBank under the indicated accession numbers.

## Supplementary Materials

The following supporting information can be downloaded at: https://www.mdpi.com/article/10.3390/v14040658/s1, Table S1: Laboratory findings on patients tested, Figure S1: Whole genome Simplot BootScan analysis including Enterovirus prototype strains; Figure S2: Maximum-likelihood phylogenetic analysis based on P1, P2 and P3 coding sequences.
