# Supplementary material for: Clinical, Laboratory, and Molecular Epidemiology of an Outbreak of Aseptic Meningitis Due to a Triple-Recombinant Echovirus in Ashburton, New Zealand
Source: Viruses. 2022 Mar 22;14(4):658. doi: 10.3390/v14040658 (PMC9030895; doi:10.3390/v14040658)
Supplement: Supplementary file 1 [file viruses-14-00658-s001.zip › viruses-1610848-supplementary.pdf]

viruses

MDPI

Supplementary materials

Clinical, Laboratory, and Molecular Epidemiology of an Outbreak of Aseptic Meningitis Due to a Triple-Recombinant Echovirus in Ashburton, New Zealand

Table S1. Laboratory findings on patients tested.

Sample	WCC ($x10^6$/L)	CSF protein (g/L)	CSF glucose (mmol/L)	Peripheral WBC ($x10^9$/L)	CRP (mg/L)
17AK4371O	243	0.32	3.2	4.6	4
17AI4233A	101	0.76	3.4	12.2	14
17AE7121N	83	0.50	3.2	4.4	11
17AE6281G	560	1.24	3.1	9.7	9
17Z79859A	216	0.76	3.3	5.0	8
17Z78549V	160	0.72	3.9	10.3	63
17WQ2027G	111	0.99	3.3	7.9	31
17WQ0610M	228	0.77	3.2	7.8	41
17Z74186F	905	0.88	3.8	13.0	10

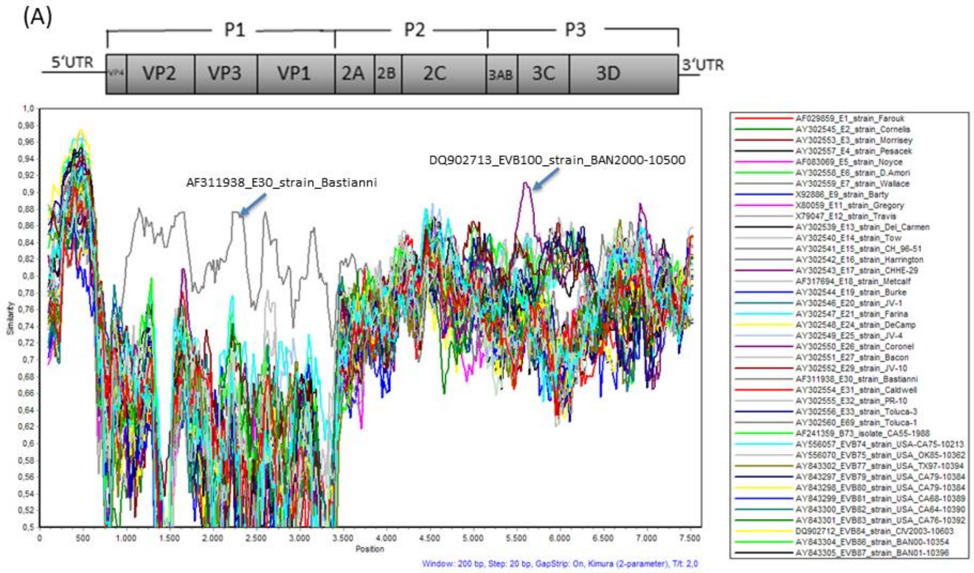

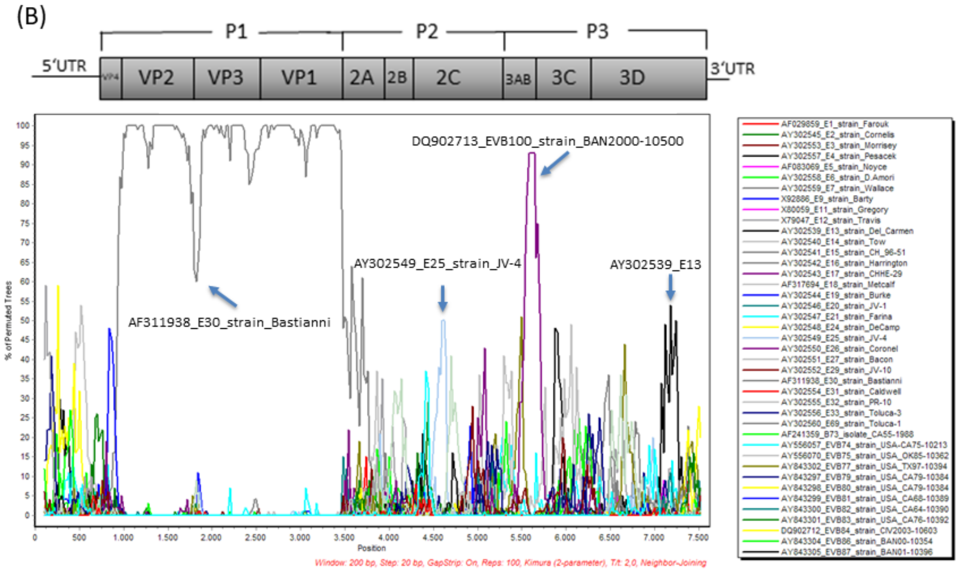

Figure S1. Whole genome Simplot BootScan analysis with the query sequence of strain NZ/Ashburton/17WQ2027G/2017-08-20 (accession number MW586892) compared to Enterovirus prototype strains mentioned in [12]. **A:** Similarity plot analysis, **B:** Bootscanning analysis on the putative recombinant strain and enterovirus prototype strains. The enterovirus genomic organization is shown in the top.

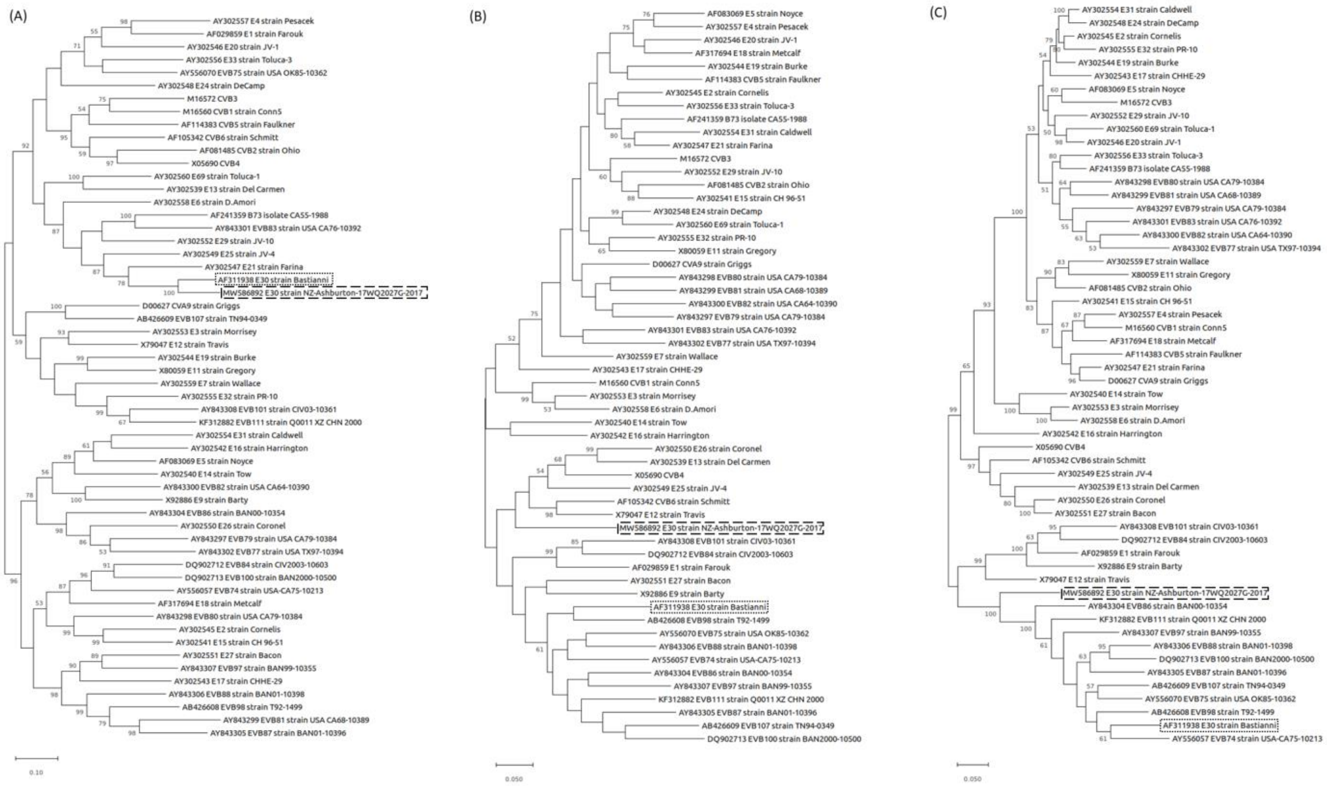

Figure S2. Maximum-likelihood phylogenetic analysis based on the P1 (**A**), P2 (**B**) and P3 (**C**) coding sequences of the recombinant NZ-Ashburton E30 enterovirus (MW58689, dashed box) and enterovirus prototype strains described in [12] including E30 prototype strain Bastianni (AF311938, dotted box).
